# First-trimester use of antiseizure medications and the risk of miscarriage: a population-based cohort study

**DOI:** 10.1136/jnnp-2023-333149

**Published:** 2024-05-22

**Authors:** Harriet Forbes, Paul Madley-Dowd, Viktor Ahlqvist, Jennifer Campbell, Neil M Davies, Rachel Liebling, Kristen Lyall, Craig Newschaffer, Jessica Rast, Torbjörn Tomson, Caichen Zhong, Cecilia Magnusson, Dheeraj Rai, Brian K Lee

**Affiliations:** 1 Epidemiology and Population Health, London School of Hygiene & Tropical Medicine, London, UK; 2 Centre for Academic Mental Health, Population Health Sciences, Bristol Medical School, University of Bristol, Bristol, UK; 3 Medical Research Council Integrative Epidemiology Unit, University of Bristol, Bristol, UK; 4 Department of Global Public Health, Karolinska Institute, Stockholm, Sweden; 5 Clinical Practice Research Datalink, Medicines and Healthcare Products Regulatory Agency, London, UK; 6 Division of Psychiatry, University College London, London, UK; 7 K.G. Jebsen Center for Genetic Epidemiology, Norwegian University of Science and Technology, Trondheim, Norway; 8 Department of Obstetrics, University Hospitals Bristol and Weston, National Health Service England, Redditch, UK; 9 Department of Epidemiology and Biostatistics, Drexel University School of Public Health, Philadelphia, Pennsylvania, USA; 10 College of Health and Human Development, The Pennsylvania State University, Pennsylvania, Texas, USA; 11 A.J. Drexel Autism Institute, Philadelphia, Pennsylvania, USA; 12 Department of Clinical Neuroscience, Karolinska Institute, Stockholm, Sweden; 13 Centre for Epidemiology and Community Medicine, Region Stockholm, Stockholm, Sweden; 14 Avon and Wiltshire Partnership NHS Mental Health Trust, Bristol, UK

**Keywords:** OBSTETRICS, PSYCHIATRY, CLINICAL NEUROLOGY

## Abstract

**Background:**

Antiseizure medications (ASMs) during the first trimester of pregnancy have been associated with an increased risk of miscarriage.

**Methods:**

We carried out a population-based cohort study using routinely collected healthcare data from the UK, 1995–2018. Pregnancies were identified in the Clinical Practice Research Datalink and we estimated the HR of miscarriage associated with prescriptions of ASMs during the first trimester of pregnancy, using Cox regression, adjusting for potential confounders, including ASM indications.

**Results:**

ASMs were prescribed during the first trimester in 7832 (0.8%) of 1 023 787 included pregnancies. 14.5% of pregnancies with first-trimester exposure to ASMs ended in miscarriage, while 12.2% without ASM exposure in the first trimester ended in miscarriage; after adjustment, there was a 1.06-fold relative hazard of miscarriage (95% CI 1.00 to 1.13) in women with first-trimester ASM use. After restricting to women with specific ASM indications, this association was not evident in women with epilepsy (adjusted HR 0.98, 95% CI 0.89 to 1.08), but was observed in women with bipolar or other psychiatric conditions (1.08, 95% CI 1.00 to 1.16) although CIs overlapped. Compared with discontinuation of ASMs prior to pregnancy, there was no evidence of increased risk of miscarriage for first-trimester ASM use in women with bipolar or other psychiatric conditions (1.02, 95% CI 0.87 to 1.20).

**Conclusion:**

We found no clear evidence to suggest that first-trimester ASM use increased the risk of miscarriage. Taken together, our analyses suggest that apparent associations between first-trimester ASM use and miscarriage may be the result of confounding by the presence of a bipolar disorder or associated unmeasured variables.

**WHAT IS ALREADY KNOWN ON THIS TOPIC**

- There is mixed evidence regarding the association between antiseizure medication (ASM) use during pregnancy and miscarriage.
- Methodological challenges exist, particularly the difficulty of separating any potential effects of ASMs from those related to the underlying condition requiring treatment (confounding by indication).
- This study aims to triangulate the evidence using multiple methods to examine whether ASM prescribing in the first trimester of pregnancy is associated with the risk of miscarriage.

**WHAT THIS STUDY ADDS**

- Our study suggests that ASM exposure during the first trimester of pregnancy is not associated with miscarriage.
- Our findings indicate previously observed associations between ASM use and miscarriage may be driven by confounding by indication.

**HOW THIS STUDY MIGHT AFFECT RESEARCH, PRACTICE OR POLICY**

- This work may help inform women considering taking ASMs during pregnancy or women currently taking ASMs at the point of a confirmed pregnancy.
- While women taking ASMs had a slightly higher incidence of miscarriage (14.5% vs 12.2% in those without ASM exposure), this work supports existing evidence suggesting ASMs are unlikely to increase the risk of miscarriage.

## Introduction

Antiseizure medications (ASMs) are used to manage epilepsy, as mood stabilisers for psychiatric conditions such as bipolar disorder and as pain relievers for conditions such as postherpetic neuralgia and migraine prophylaxis. Between 6 and 34 in 1000 pregnant women use ASMs during pregnancy, with the numbers rising over time.^1 2^ The use of ASMs during pregnancy must balance the beneficial effects of treatment on disease management, such as preventing seizures or bipolar relapses, with the potential for teratogenic effects of in-utero drug exposure on the developing fetus. Pregnancy is a risk factor for discontinuation of ASMs,^1 3^ despite discontinuation posing serious risks to both mother and child.^4^ As such, it is essential to accurately estimate both risks or risk reductions of adverse pregnancy outcomes such as miscarriage that may be associated with ASM use during pregnancy, to best inform clinical management decisions.

There is now consensus that the use of certain ASMs during pregnancy leads to an increased risk of congenital malformations in offspring.^1^ During pregnancy, the developing fetus is exposed to ASMs in utero through the placental transport of drugs, however the pathophysiological mechanisms associated with their teratogenicity have not been fully understood. The risk of miscarriage after the use of ASMs in pregnancy remains uncertain, with some studies suggesting ASMs increase the risk of miscarriages,^5^ while others do not.^6–9^ Studies investigating drug-specific effects have also reported inconsistent results.^10–12^ Research in this area is hampered by methodological challenges, particularly the difficulty of separating any potential effects of ASMs from those related to the underlying condition requiring treatment (confounding by indication). Furthermore, as ASMs are not widely used in the general population, many studies have lacked adequate power to investigate the risks associated with specific ASMs. Studies enrolling pregnant women have also suffered from gestational age bias, where early pregnancy losses are better captured for ASM-exposed women, as they are recruited earlier in pregnancy; this may overestimate any increased risk of early pregnancy loss in women taking ASMs. Furthermore, much of the research carried out to date has lacked appropriate control for potentially important confounders, such as obesity and smoking during pregnancy.

To address these gaps, this study aims to triangulate the evidence using multiple methods, including conducting indication-based analysis and an active comparator design, to examine whether ASM prescribing in the first trimester of pregnancy is associated with the risk of miscarriage.

## Methods

We conducted a population-based cohort study among women with a pregnancy within the Clinical Practice Research Datalink (CPRD) Pregnancy Register.

### Data sources

We used data from the CPRD GOLD version, the CPRD Pregnancy Register, the Hospital Episode Statistics database (HES), Office for National Statistics (ONS) death certificate data and Index of Multiple Deprivation (IMD) data.

CPRD GOLD holds de-identified primary care data from ~9% of the UK population and is approximately representative of the UK population in terms of age and sex.^13^ Individual-level patient data are available since registration at the general practice, including diagnoses (recorded using a coded thesaurus of clinical term, known as Read codes),^14^ prescriptions (recorded using British National Formulary codes) and demographic data.

HES data cover ~80% of English practices included in CPRD and contain all National Health Service-funded hospital admissions, outpatient records, maternity records and procedures, in England since 1997 (or 2003 for outpatient records). HES data include diagnoses (coded using The International Classification of Diseases version 10 (ICD-10) and the Operating Procedure Codes Supplement version 4 (OPCS-4)) but not hospital-based prescriptions.^9^ IMD data provide area-level data for all CPRD patients by mapping patients’ home postcode (or general practice postcodes if home postcode is missing) to geographical areas with predefined deprivation scores; data from several indicators, covering a range of economic, social and housing issues, are combined into a single deprivation score. ONS data provide the date and cause of death, from 1998, for patients registered in general practices in England and Wales.

The CPRD Pregnancy Register lists and characterises all pregnancies identified in CPRD GOLD for women aged 11–49 years, based on an algorithm that uses data from the primary care record only.^15^ A single record represents a unique pregnancy and women can have multiple pregnancies. The register includes pregnancy outcome (where available) and estimated pregnancy timings, including pregnancy start date (ie, date of conception) and pregnancy end date. These variables were derived using coded data on the last menstrual period (LMP) and a variety of other pregnancy-related codes in CPRD GOLD.

### Study design and population

We included pregnancies starting between 1 January 1995 and 31 December 2018, among women registered with an ‘up to standard’ practice (the date at which data in the practice is considered to have continuous high-quality data fit for use in research) for ≥365 consecutive days prior to pregnancy start (to ensure sufficient time to record baseline characteristics and use of ASMs prior to pregnancy) and registered until pregnancy end. We also ensured pregnancy start was at least 9 months before the last data collection date for that practice, to allow for attainment of outcomes. Where pregnancy outcome was unknown, we searched for the outcome in linked HES data and where pregnancies were conflicted we applied an algorithm to identify real and historical pregnancies (online supplemental methods S1).^16^ To maximise study power, in our main analysis we included patients with and without linked HES data. Multiple pregnancies were excluded, as pregnancy loss is considerably higher in these pregnancies. We also excluded women with missing age.

10.1136/jnnp-2023-333149.supp1 Supplementary data

### Outcome

Miscarriages were identified through the CPRD Pregnancy Register algorithm from Read codes^15^ in the primary care record. Miscarriages included pregnancies with a blighted ovum (an older term for a certain type of pregnancy that leads to an early miscarriage). Where the Pregnancy Register recorded the pregnancy outcome as unknown, we searched for ICD-10 codes indicating a miscarriage in linked HES data (see online supplemental methods S1 for more details).

### Exposure

We identified ASM prescriptions from primary care records from 365 days prior to pregnancy start up until the pregnancy end. ASM prescriptions included all those with Anatomical Therapeutic Chemical codes N03A (antiseizure drugs) and N05BA09 (clobazam). We identified the start and end of each prescription (online supplemental methods S2) to identify women with some exposure during the first trimester. Our primary exposure was using one or more ASMs during the first trimester of pregnancy; the first trimester was defined as the first 90 days from pregnancy start date. Unexposed pregnancies were those without ASM exposure in the first trimester. The daily dose in milligrams was calculated for each prescription by multiplying the number of tablets taken per day by the dose per tablet. We further classified ASM exposure in the first trimester by daily dose (low, medium or high; derivation of cut-offs described in Methods S2) and polytherapy or monotherapy. Monotherapies were classified as lamotrigine, valproate, carbamazepine, pregabalin, levetiracetam, gabapentin, phenytoin, topiramate, clonazepam or other.

### Antiseizure medication indications

We identified the following indications, prior to pregnancy start: epilepsy, bipolar disorder and other psychiatric conditions (generalised anxiety disorder, depression and schizophrenia) and other somatic conditions (neuropathic pain, restless leg syndrome and migraine) (online supplemental methods S3). Each patient could have multiple indications and thus contribute to several of our analyses.

### Covariates

Maternal characteristics derived at pregnancy start date included; age in years (<18, 18–24, 25–29, 30–34, ≥35), socioeconomic position (IMD score), ethnicity (white or other than white, derived from CPRD and HES inpatient data), problem drinking, illicit drug use, body mass index, co-prescriptions of antidepressants and antipsychotics, gravidity, history of miscarriage and year of pregnancy start (1995–2000, 2001–2005, 2006–2010, 2011–2015, 2016–2018).

### Follow-up time

Follow-up began at the pregnancy start date. If ASMs were started during the first trimester (ie, the woman took no ASM in the year prior to pregnancy), a woman contributed time from pregnancy start to 1-day prior to drug prescription start date to the unexposed group and time to the exposed group from date of ASM prescription start date. Follow-up ended at the earliest of: miscarriage or other loss (including ectopic or molar pregnancies, voluntary terminations and unspecified losses), study end (31 December 2018) or gestational age of 24 weeks (losses after 24 weeks are classified as stillbirths in the UK).

### Statistical analysis

#### Main analysis

First, we described baseline characteristics of the cohort by exposure status at pregnancy start and rates of miscarriage by ASM indication. We then calculated the proportion of women experiencing miscarriage and the crude and adjusted HRs of miscarriage using Cox proportional hazards (with gestational age as the underlying timescale) with robust SEs to account for women contributing to several pregnancies. Maternal age, year of pregnancy start, IMD, ASM indication, history of miscarriage and ethnicity were considered as covariates. We first analysed all identified pregnancies, and then restricted to women with each ASM indication separately. The proportional hazards assumption was explored by testing for a zero slope in the scaled Schoenfeld residuals.

#### Discontinuers and active comparator

In order to make the exposed and unexposed more comparable, we changed the comparator groups. First, we compared ASM users in the first trimester with ‘pre-pregnancy discontinuers’, who used ASMs in the 6 months prior to pregnancy, but did not use them in the first trimester. Second, we estimated the risk of miscarriage in non-lamotrigine ASM users compared with lamotrigine users. This active-comparator design increases the overlap of unmeasured characteristics between the groups to reduce the potential for unmeasured confounding. We chose lamotrigine as it is the most commonly prescribed ASM with the safest known risk profile.

#### Discordant pregnancy exposure

To account for unmeasured confounding from genetic and environmental factors that remain constant within the same woman, we examined women with ≥2 pregnancies with discordant ASM use to estimate a within-individual effect that accounts for confounding shared between pregnancies in the same mother.^17 18^ We performed a stratified Cox regression analysis with robust variance estimation, including a separate stratum for each woman. As in the main analysis, we adjusted for IMD, year of pregnancy start, maternal age and ASM indications, but not history of miscarriage.

#### Dose and polytherapy

To investigate whether any associations had a dose-response effect, among first-trimester ASM users, we investigated whether women on medium or high ASM dose (compared with low dose, overall and for the four most common ASMs) and women using ASM polytherapy (compared with monotherapy), had a higher risk of miscarriage.

#### Sensitivity analyses

Sensitivity analyses to test the robustness of our findings to other biases and measurement error are described in online supplemental methods S4.

### Patient and public involvement

All analyses used pre-existing data and, as such, neither patients nor the public was involved in the study design, data collection and analysis, interpretation of findings, decision to publish or preparation of the manuscript.

All code lists can be provided on request.

## Results

**Figure 1:**
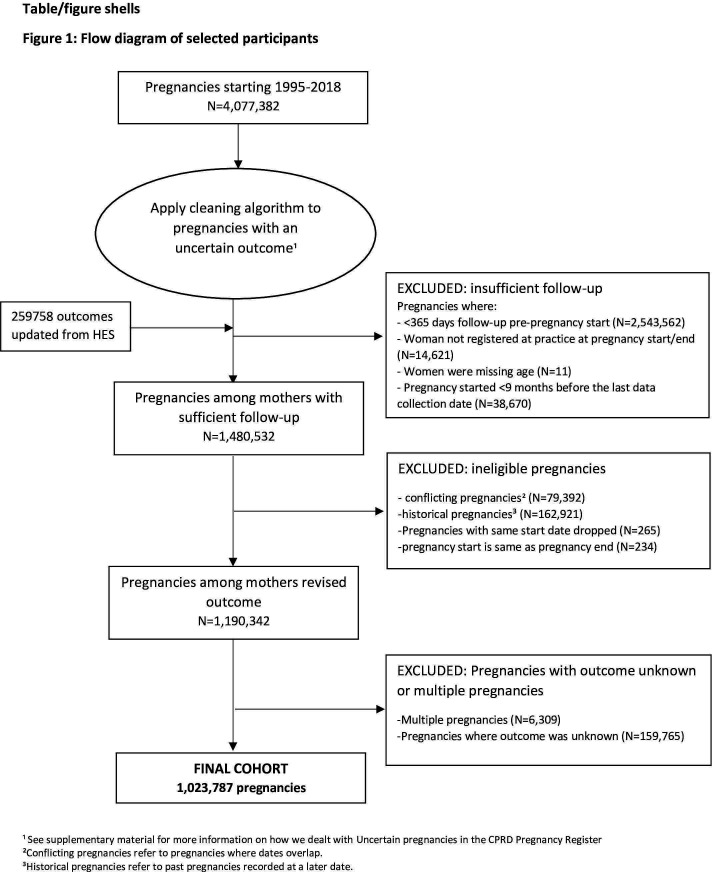
Flow diagram of selected participants. ¹See online supplemental material for more information on how we dealt with uncertain pregnancies in the Clinical Practice Research Datalink Pregnancy Register. ²Conflicting pregnancies refer to pregnancies where dates overlap. ³Historical pregnancies refer to past pregnancies recorded at a later date. HES, Hospital Episode Statistics.

Of the 1 190 343 pregnancies in mothers with eligible follow-up, we excluded 6309 (0.5%) multiple pregnancies and 159 765 (13.4%) pregnancies with an unknown outcome, resulting in 1 023 787 pregnancies among 661 297 women (figure 1). There were 125 424 (12.3%) miscarriages, 134 336 (13.1%) voluntary terminations, 10 681 (1.0%) ectopic pregnancies, 945 (0.1%) molar pregnancies and 6867 (0.7%) unspecified losses.

In total, 12.23% with no ASM exposure had a miscarriage, while 14.54% with first-trimester ASM exposure had a miscarriage. Women with each ASM indication, regardless of ASM use, had an increased risk of miscarriage (table 1). Women with epilepsy had 1.07 times higher risk (HR 1.07, 95% CI 1.02 to 1.12), while women with other somatic conditions (1.06, 95% CI 1.05 to 1.08) and women with bipolar or other psychiatric conditions (1.11, 95% CI 1.10 to 1.13) exhibited similar increases in risk as well.

**Table 1:** Association between each indication for ASMs and miscarriage, in the whole cohort (N=1 023 787)

Indication	N miscarriages	Total pregnancies	Per cent	Unadjusted HR (95% CI)	Fully adjusted* HR (95% CI)
No epilepsy	123 669	1 010 553	12.24	1.00 (ref)	1.00 (ref)
Epilepsy	1755	13 234	13.26	1.09 (1.04 to 1.15)	1.07 (1.02 to 1.12)
No bipolar or other psychiatric conditions	75 415	653 744	11.54	1.00 (ref)	1.00 (ref)
Bipolar or other psychiatric conditions	50 009	370 043	13.51	1.18 (1.17 to 1.20)	1.11 (1.10 to 1.13)
No somatic conditions	106 901	889 312	12.02	1.00 (ref)	1.00 (ref)
Somatic conditions	18 523	134 475	13.77	1.15 (1.13 to 1.17)	1.06 (1.05 to 1.08)

*Adjusted for: maternal age, year of pregnancy start, IMD, history of pregnancy loss, other ASM indication.

ASM, antiseizure medications ; IMD, Index of Multiple Deprivation.

In total, 7832 pregnancies (0.8%) were exposed to ASMs during the first trimester (table 2). Of the 13 234 women with epilepsy, 4725 (35%) were exposed to ASMs during the first trimester. First-trimester ASM users were older, lived in more deprived neighbourhoods, more likely to be a smoker, be obese, have problem drinking and illicit drug use and be on other prescription medication (specifically antipsychotics and antidepressants). Among first-trimester users, 4725 (60.3%) had epilepsy, 5002 (63.9%) had bipolar or another psychiatric condition indicated for ASMs and 2313 (29.5%) had another somatic condition indicated for ASMs (women could have multiple indications).

**Table 2:** Maternal characteristics at the start of pregnancy, by ASM exposure status

Variable		Total	Unexposed to ASMs in first trimester, n (%)	First-trimester ASM exposure, n (%)
		1 023 787 (100.0)	1 015 955 (100.0)	7832 (100.0)
Age in years	<18	38 216 (3.7)	38 055 (3.7)	161 (2.1)
	18–24	231 835 (22.6)	230 299 (22.7)	1536 (19.6)
	25–29	265 563 (25.9)	263 527 (25.9)	2036 (26.0)
	30–34	285 357 (27.9)	283 181 (27.9)	2176 (27.8)
	≥35	202 816 (19.8)	200 893 (19.8)	1923 (24.6)
ASM indication* (women could have multiple indications, thus contribute to several groups)	Epilepsy	13 234 (1.3)	8509 (64.3)	4725 (35.70)
	Bipolar and other psychiatric	370 043 (36.1)	365 041 (98.7)	5002 (1.3)
	Other somatic	134 475 (13.1)	132 162 (98.3)	2313 (1.7)
	No recorded indication	587 261 (57.4)	587 053 (99.9)	208 (0.1)
IMD quintile	1 (least deprived)	194 057 (19.0)	192 854 (19.0)	1203 (15.4)
	2	178 762 (17.5)	177 569 (17.5)	1193 (15.2)
	3	196 900 (19.2)	195 515 (19.2)	1385 (17.7)
	4	208 567 (20.4)	206 804 (20.4)	1763 (22.5)
	5 (most deprived)	245 501 (24.0)	243 213 (23.9)	2288 (29.2)
Ethnicity	White	645 744 (63.1)	640 775 (63.1)	4969 (63.4)
	South Asian	32 858 (3.2)	32 708 (3.2)	150 (1.9)
	Black	17 869 (1.7)	17 792 (1.8)	77 (1.0)
	Other	11 580 (1.1)	11 514 (1.1)	66 (0.8)
	Mixed	6950 (0.7)	6911 (0.7)	39 (0.5)
	Not stated	308 786 (30.2)	306 255 (30.1)	2531 (32.3)
Year of pregnancy start	1995–2000	136 079 (13.3)	135 396 (13.3)	683 (8.7)
	2001–2005	245 206 (24.0)	243 880 (24.0)	1326 (16.9)
	2006–2010	305 563 (29.8)	303 521 (29.9)	2042 (26.1)
	2011–2015	249 123 (24.3)	246 697 (24.3)	2426 (31.0)
	2016–2018	87 816 (8.6)	86 461 (8.5)	1355 (17.3)
Smoking status	Non-smoker	415 837 (40.6)	413 174 (40.7)	2663 (34.0)
	Current smoker	307 607 (30.0)	304 712 (30.0)	2895 (37.0)
	Ex-smoker	245 838 (24.0)	243 786 (24.0)	2052 (26.2)
	Not stated	54 505 (5.3)	54 283 (5.3)	222 (2.8)
BMI	Underweight	32 972 (3.2)	32 729 (3.2)	243 (3.1)
	Normal weight	463 975 (45.3)	461 062 (45.4)	2913 (37.2)
	Overweight	239 081 (23.4)	237 260 (23.4)	1821 (23.3)
	Obese	182 915 (17.9)	180 703 (17.8)	2212 (28.2)
	Not stated	104 844 (10.2)	104 201 (10.3)	643 (8.2)
Problem drinking		10 176 (1.0)	10 017 (1.0)	159 (2.0)
Illicit drug use		2294 (0.2)	2182 (0.2)	112 (1.4)
Primary care consultations†	0	83 181 (8.1)	82 986 (8.2)	195 (2.5)
	1–3	271 949 (26.6)	271 424 (26.7)	525 (6.7)
	4–10	439 136 (42.9)	436 788 (43.0)	2348 (30.0)
	>10	229 521 (22.4)	224 757 (22.1)	4764 (60.8)
Other prescriptions†	Antipsychotics	808 (0.1)	754 (0.1)	54 (0.7)
	Antidepressants	114 288 (11.2)	111 534 (11.0)	2754 (35.2)
	Multivitamins	603 (0.1)	591 (0.1)	12 (0.2)
	Folic acid	268 760 (26.3)	263 859 (26.0)	4901 (62.6)
Previous miscarriage		100 302 (9.8)	99 482 (9.8)	820 (10.5)

*Bipolar disorder and other psychiatric conditions (generalised anxiety disorder, depression and schizophrenia), other somatic conditions (neuropathic pain, restless leg syndrome and migraine).

†In year before pregnancy.

ASM, antiseizure medication; BMI, body mass index; IMD, Index of Multiple Deprivation.

### Main analysis

**Figure 2:**
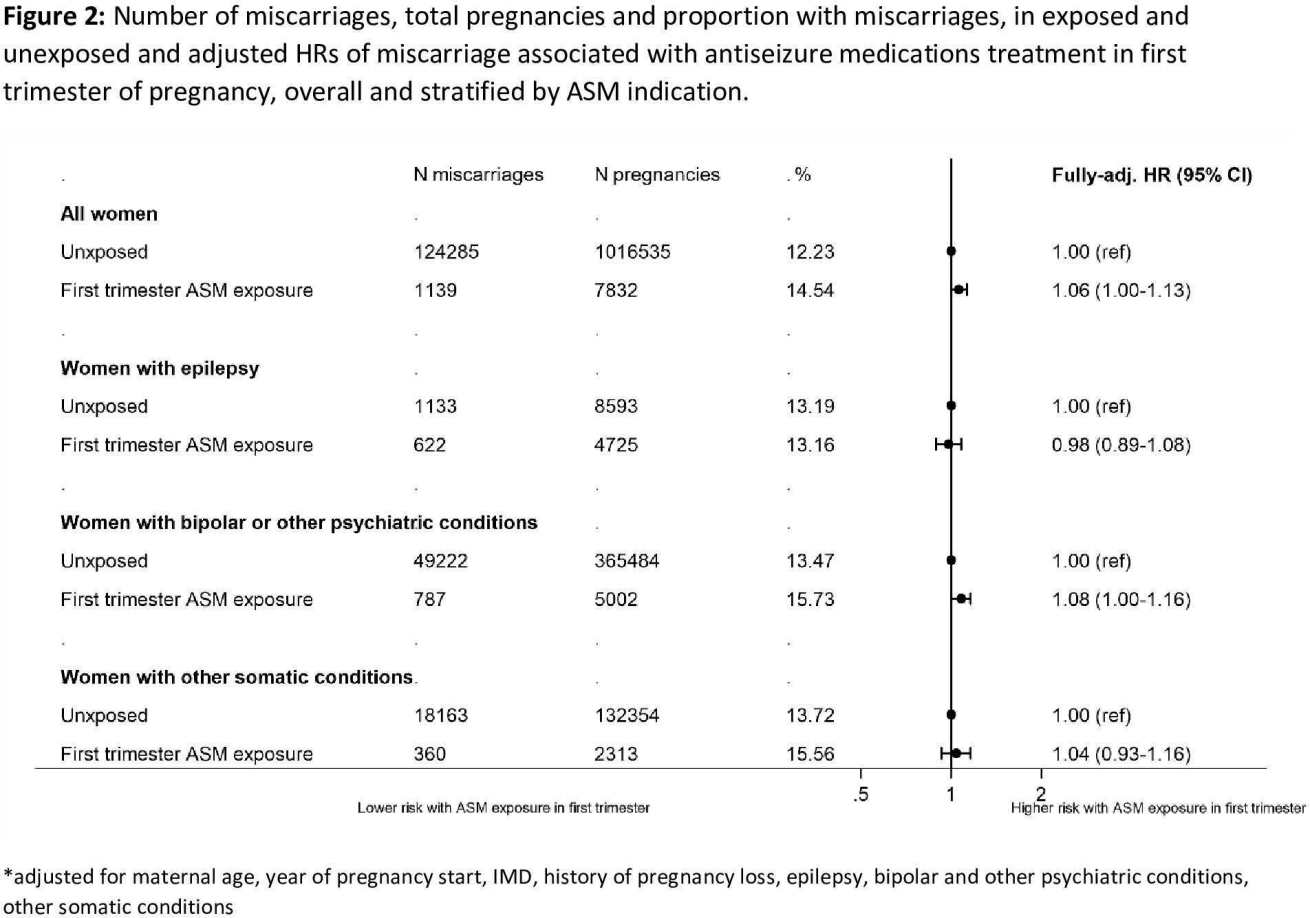
Number of miscarriages, total pregnancies and proportion with miscarriages, in exposed and unexposed and adjusted HRs of miscarriage associated with antiseizure medications treatment in the first trimester of pregnancy, overall and stratified by ASM indication. *Adjusted for maternal age, year of pregnancy start, Index of Multiple Deprivation, history of pregnancy loss, epilepsy, bipolar and other psychiatric conditions, other somatic conditions. ASM, antiseizure medication.

In total, among pregnancies with first-trimester exposure to ASMs, 14.5% ended in miscarriage, while those without ASM exposure in the first trimester experienced 12.2% (figure 2 and online supplemental table S1). After adjustment (figure 2), there was evidence of a 1.06-fold increased risk of miscarriage among those exposed to ASMs in the first trimester, compared with ASM unexposed (HR 1.06, 95% CI 1.00 to 1.13). After stratifying on indication (figure 2 and online supplemental table S1) there was an association among women with bipolar or other psychiatric conditions (1.08, 95% CI 1.00 to 1.16), but not women with epilepsy (both exposed and unexposed experienced 13.2% miscarriage, 0.98, 95% CI 0.89 to 1.08) or other somatic conditions (1.04, 95% CI 0.93 to 1.16). Adjusting for ethnicity did not materially affect the results (online supplemental table S1).

### Discontinuers and active comparator

**Figure 3:**
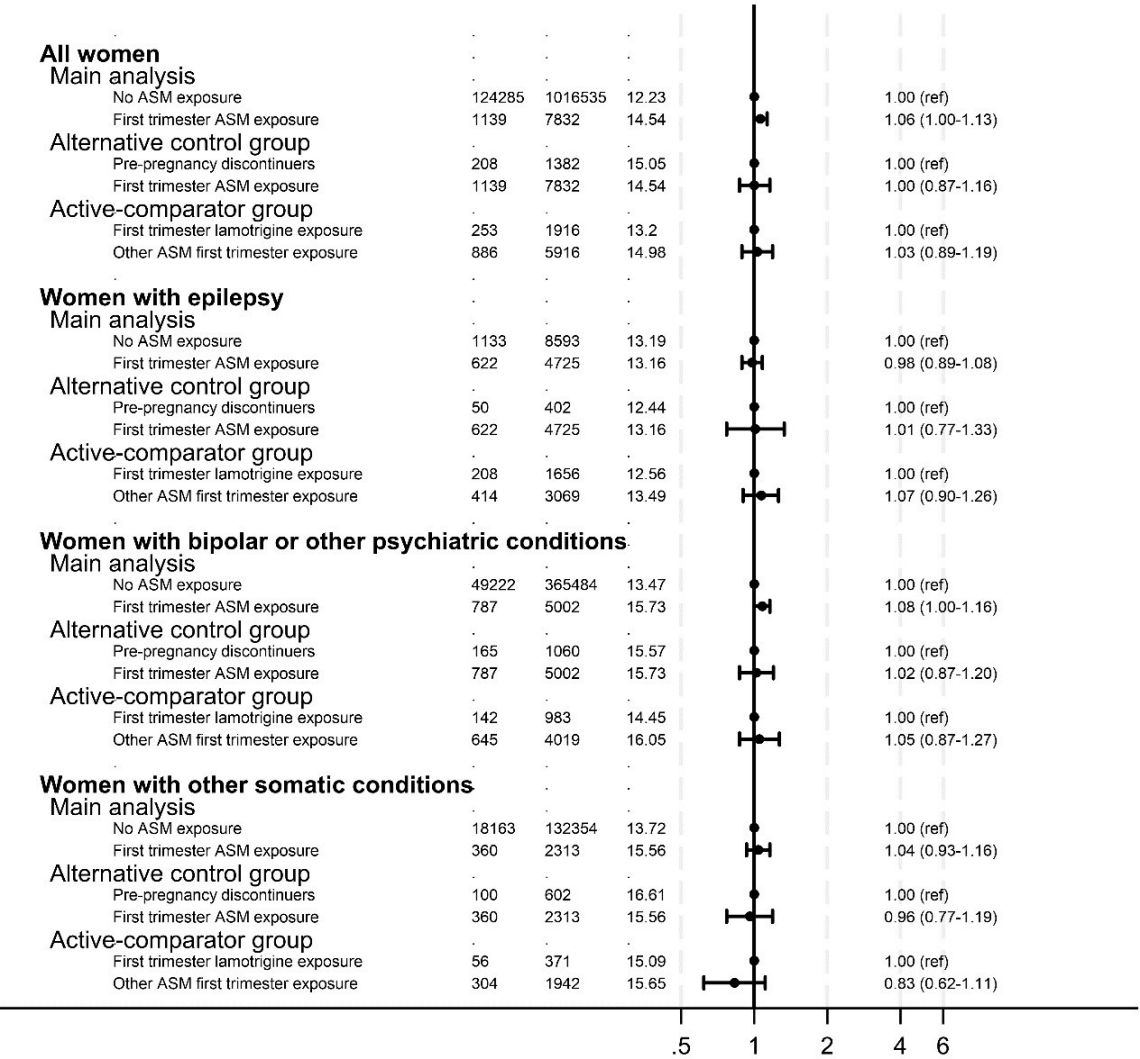
Association between first-trimester ASM use and miscarriage: results from different analytical methods. Adjusted for maternal age, year of pregnancy start, Index of Multiple Deprivation, epilepsy, bipolar and other psychiatric conditions, other somatic conditions and for analyses excluding the discordant ASM pregnancies, history of pregnancy loss. ASM, antiseizure medication.

In the main analysis, ASM exposed women were compared with all individuals without ASM exposure (N=1 016 535). We also compared first-trimester ASM users to two different control groups, specifically pre-pregnancy discontinuers (N=1382 discontinuers) and lamotrigine users (N=1916 lamotrigine users); in all these analyses, first-trimester ASM exposure was not associated with miscarriage risk, including within women with bipolar or other psychiatric conditions (figure 3).

### Discordant pregnancy exposure

In the analysis of discordant exposure pregnancies within the same women, pregnancies when the mothers were exposed to ASMs in the first trimester (N=2039) had a greater risk of miscarriage, compared with pregnancies where the women were unexposed (N=3216) (1.28, 95% CI 1.10 to 1.49). We explored whether this could be explained by the order of the pregnancy exposed to ASMs, by dividing the exposure-discordant pregnancy-pairs into two subgroups defined by whether the first or second pregnancy was exposed. An increased risk of miscarriage was found when the first pregnancy was exposed and the second pregnancy was unexposed, but not when the first pregnancy was unexposed and the second pregnancy exposed (see online supplemental table S2). Thus, the presence of an association in the discordant pregnancy analysis could be explained by the ordering of the pregnancy and not necessarily the result of the ASM itself.

### Dose and polytherapy

**Figure 4:**
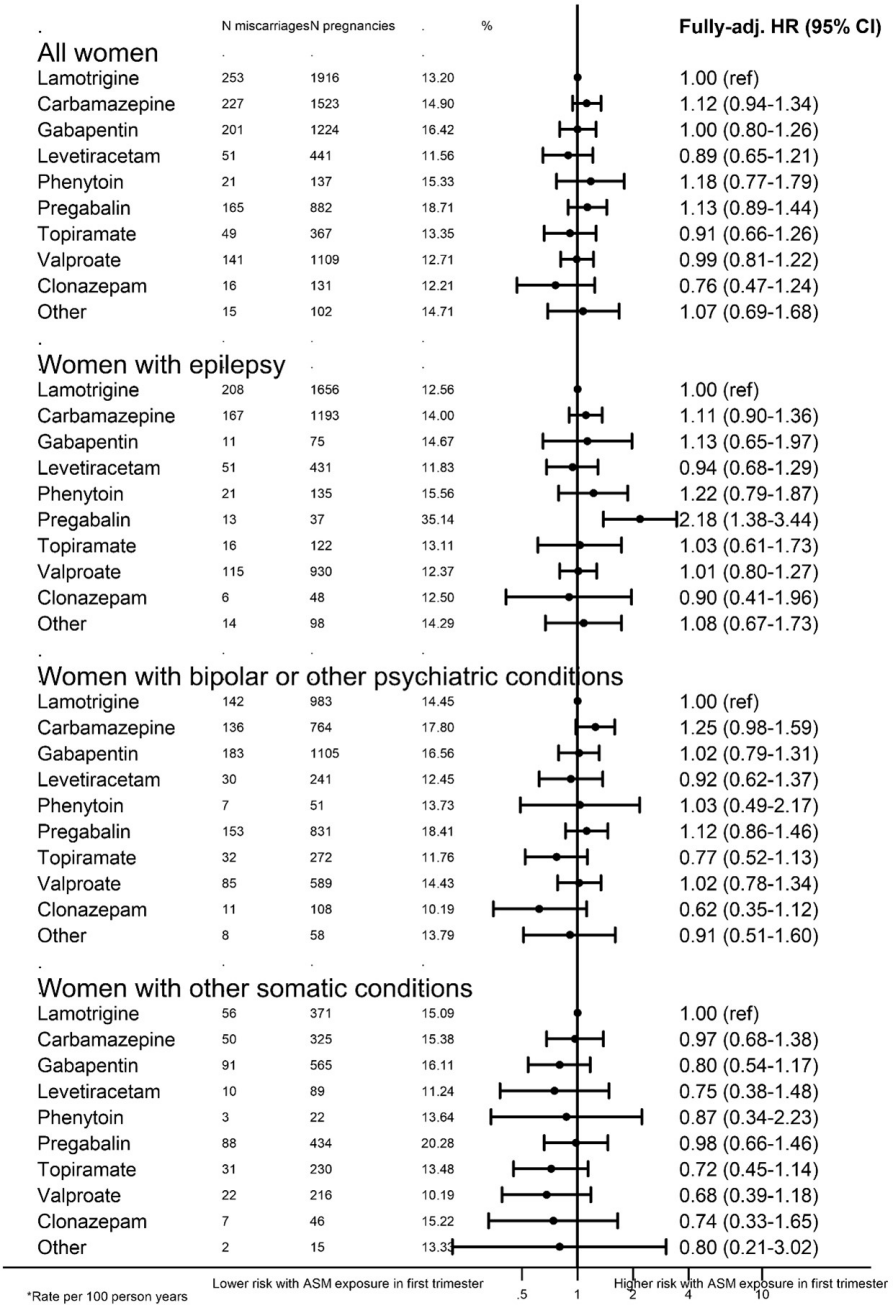
HRs of miscarriage associated with specific antiseizure medications in pregnancy, among women with first-trimester ASM exposure, compared with lamotrigine exposure. *Adjusted for: maternal age, year of pregnancy start, Index of Multiple Deprivation, history of pregnancy loss, epilepsy, bipolar, other psychiatric conditions, other somatic conditions. ASM, antiseizure medication.

Women with a high ASM dose in the first trimester had an increased risk of miscarriage to those who received a low dose (table 3), (1.12, 95% CI 0.96 to 1.30). After restricting on indication, this was only observed among women with epilepsy (association of high vs low dose; 1.33, 95% CI 1.06 to 1.66). ASM-specific results for the four most common ASMs (see online supplemental table S3) shows that among women with epilepsy taking valproate, those on high dose were 1.7 times (95% CI 1.00 to 2.96) as likely to have a miscarriage compared with those on a low dose. However, this analysis was limited by small numbers. Polytherapy was not associated with the risk of miscarriage, compared with women taking monotherapy in the first trimester (table 3). We analysed the relative risks of miscarriage associated with specific ASMs in pregnancy, among women with first-trimester ASM exposure (figure 4), compared with lamotrigine exposure. In the whole cohort, no single ASM was associated with an increased risk of miscarriage, though the CIs were wide. Among women with epilepsy, pregabalin was associated with a twofold increased risk of miscarriage, compared with lamotrigine users (2.18, 1.38 to 3.44), though only 37 pregabalin users contributed to this result and the association was not found for other indications.

**Table 3:** Number (and percentage) of events and adjusted HRs for miscarriage to the dose during pregnancy, among those with any antiseizure medications treatment in the first trimester

			N events	%	Unadjusted HR	Fully adjusted HR* (95% CI)
All	Dose	Low	328	14.1	1.00 (ref)	1.00 (ref)
		Medium	538	14.3	1.02 (0.89 to 1.17)	1.03 (0.90 to 1.17)
		High	273	15.9	1.13 (0.96 to 1.32)	1.12 (0.96 to 1.30)
	Polytherapy or monotherapy	Monotherapy	987	14.6	1.00 (ref)	1.00 (ref)
		Polytherapy	152	13.9	0.95 (0.80 to 1.12)	1.01 (0.85 to 1.19)
Epilepsy	Dose	Low	133	11.1	1.00 (ref)	1.00 (ref)
		Medium	327	13.4	1.23 (1.00 to 1.51)	1.19 (0.98 to 1.45)
		High	162	15	1.37 (1.09 to 1.73)	1.33 (1.06 to 1.66)
	Polytherapy or monotherapy	Monotherapy	484	12.9	1.00 (ref)	1.00 (ref)
		Polytherapy	138	14.3	1.12 (0.93 to 1.34)	1.09 (0.91 to 1.30)
Bipolar and other psychiatric	Dose	Low	238	15.7	1.00 (ref)	1.00 (ref)
		Medium	351	15.3	0.98 (0.83 to 1.15)	0.97 (0.83 to 1.13)
		High	198	16.7	1.06 (0.88 to 1.27)	1.04 (0.87 to 1.24)
	Polytherapy or monotherapy	Monotherapy	695	15.8	1.00 (ref)	1.00 (ref)
		Polytherapy	92	15.1	0.95 (0.77 to 1.17)	1.01 (0.82 to 1.25)
Other somatic	Dose	Low	116	16	1.00 (ref)	1.00 (ref)
		Medium	153	14.6	0.92 (0.73 to 1.16)	0.94 (0.74 to 1.18)
		High	91	16.9	1.05 (0.80 to 1.37)	1.05 (0.81 to 1.37)
	Polytherapy or monotherapy	Monotherapy	319	15.6	1.00 (ref)	1.00 (ref)
		Polytherapy	41	15	0.94 (0.69 to 1.29)	1.06 (0.76 to 1.46)

*Adjusted for: maternal age, year of pregnancy start, Index of Multiple Deprivation, history of pregnancy loss, other indications (epilepsy, bipolar or other psychiatric conditions and other somatic conditions) and consultation rate.

### Sensitivity analyses

When separating prevalent and new users (online supplemental table S4), the increased risk of miscarriage among women with bipolar and other psychiatric conditions was greater in new than prevalent users. When restricting to patients with linked HES data, first pregnancies and when requiring two prescriptions in the first trimester to be classified as exposed, the results were similar (online supplemental table S4). When comparing the women included in the study to those excluded because of missing pregnancy outcome, there were no major differences between the two groups (online supplemental table S5).

## Discussion

In this large population-based cohort study, we found no clear evidence to suggest that first-trimester ASM use increased the risk of miscarriage. First-trimester ASM use appeared to be associated with a very small increased risk of miscarriage, compared with women without first-trimester ASM exposure (HR 1.06, 95% CI 1.00 to 1.13), however after restricting to women with specific ASM indications, this small association was observed only in women with bipolar or other psychiatric conditions, not women with epilepsy or other somatic conditions. The association was interrogated further by altering the comparison group to be pre-pregnancy discontinuers, who may be more similar demographically/in indication characteristics to ASM users; no association was found in women with bipolar disorder using this comparator group, suggesting that the previous association may be explained by confounding from the underlying disorder, rather than ASM drug exposure, a bias known as confounding by indication. Taken together, our analyses suggest that apparent associations between first-trimester ASM use and miscarriage may be the result of confounding by the presence of a bipolar disorder or residual confounding from unmeasured variables.

Studies investigating the association between ASM use during pregnancy and pregnancy loss have found conflicting results. A systematic review of studies up to 2015 found ASMs exposure in pregnancy was not associated with miscarriage (three studies, 10 327 pregnancies, OR 1.30, 0.61 to 2.79, I^2^ 32%), among women with epilepsy.^6^ The largest study in the review was a general population-based Danish registry study, among almost 1 million pregnancies, of which 4700 (0.5%) were exposed to ASMs; this study found a small increased risk of miscarriage associated with ASM use during pregnancy (risk ratio 1.13, 95% CI 1.04 to 1.24). However, the authors concluded this finding was driven by confounding by indication as the association was only observed in women without an epilepsy diagnosis.^19^ Since the 2015 systematic review, three relevant studies have been published. A recent study using administrative data from New Zealand, among 2728 ASM users and 469 816 ASM non-users, found a 1.4-fold increased risk of miscarriage in ASM users; however, this study only accounted for maternal age and had no information on the underlying indication.^9^ An Italian study using hospital-based miscarriage data among 145 243 pregnancies found no association between ASM use and miscarriage^8^ and another study using data from a North American ASM registry found after accounting for gestational age at enrolment, there was no evidence of an association between ASM use and miscarriage.^7^ Overall, the existing evidence is in line with our findings pointing toward no association between first trimmest ASM use and miscarriage.

The population-based Danish registry study looked at 898 exposure-discordant pregnancies within the same women with epilepsy, and found a slightly reduced risk of miscarriage (0.83, 0.69 to 1.00) for exposed compared with unexposed pregnancies.^19^ By contrast, in our exposure-discordant pregnancies analysis among 5278 pregnancies, we showed an increased risk of miscarriage among our exposed group, compared with the unexposed. This may reflect different patterns of exposure in consecutive pregnancies. A miscarriage in the first pregnancy may increase the chance of a woman stopping ASM medication in the second pregnancy; we observed a substantially elevated HR for miscarriage when the first pregnancy was exposed to ASMs, but not when the first pregnancy was unexposed.

A network meta-analysis suggested that valproate, primidone and topiramate are associated with an increased risk of fetal loss (a combined variable of any pregnancy loss), compared with no ASM exposure.^20^ In our study, we chose an active-comparator control, to provide information to clinicians about the risks of outcomes relative to lamotrigine which is generally considered the safest alternative. While this design may be subject to confounding by indication, as certain medications may be given to patients with greater disease severity. We found women exposed to any ASM had similar risks of miscarriage, compared with lamotrigine. An earlier study by Mostacci *et al*,^21^ found that of 30 pregnancies exposed to pregabalin, 23.3% ended in spontaneous abortion versus 11.3% in unexposed, resulting in an unadjusted OR of 2.39, 95% CI 0.87 to 5.75. The present study found evidence of an adjusted association of pregabalin with miscarriage, but only for women with epilepsy (n=37). Because the association was present only in a small subgroup, and there was no association of pregabalin in other indication subgroups nor in the overall group of all women who used pregabalin (n=882), we would caution against over-interpretation of this potential signal. Nevertheless, additional data regarding pregabalin and miscarriage would be useful. Our results were broadly in line with data from the European and International Registry of Antiepileptic Drugs in Pregnancy, a prospective observational study of 7055 pregnancies among women with epilepsy on ASMs, which found intrauterine death was not associated with ASM type.^22^

We found evidence of a potentially increased risk of miscarriage, associated with taking high-dose ASMs compared with low-dose ASMs, only in women with epilepsy; in further analysis of specific ASMs, only valproate showed a dose-response effect. A large Danish registry-based study also found women with and without epilepsy on high-dose ASMs had a greater risk of miscarriage; however, this study compared women on high dose to women not taking ASMs, potentially suffering from confounding.^19^ By contrast EURAP data, among 7055 ASM-exposed pregnancies in women with epilepsy, found the risk of intrauterine death was not associated with dose, when analysing lamotrigine, valproate and carbamazepine separately.^22^ A greater risk of miscarriage with higher ASM dose may suggest the presence of a threshold effect or it may reflect higher doses being given to women with greater disease severity, and severity of indication being the key confounder. As a dose-response effect was only observed among women with epilepsy, this suggests that confounding by severity may best explain these findings. Higher ASM doses in women with epilepsy may reflect poor seizure control, which is a known risk factor for miscarriage, not reliably recorded in CPRD and its linked data sets.

The main strengths of our study are the use of a large, general population sample of clinically recognised pregnancies, over a 22-year study period, ensuring our main analysis was well-powered with high external validity. This study is unique in assessing the association among women with non-epilepsy indications for ASMs, helping us untangle the role of underlying indication versus the drug effects on miscarriage risk. Validation work comparing the CPRD Pregnancy Register against linked hospital maternity records indicates has demonstrated the CPRD Pregnancy Register has high sensitivity (77%) in identifying hospital-based early pregnancy losses, suggesting most pregnancies are well captured in the register; furthermore, previous work showed the overall miscarriage rates in the Pregnancy Register (of 12%–13%) compare favourably with estimates from external sources.^11^ Finally, by using a series of analytical methods we have been able to explore whether observational associations are likely to reflect causal effects, thereby strengthening the relevance and applicability of our findings.

While our study has several strengths, it also has some limitations. First, there is potential for selection bias related to the ascertainment of pregnancies. The CPRD Pregnancy Register only records pregnancies reported to primary care services. Many pregnancies end in miscarriage before the pregnancy is clinically recognised; if preclinical losses are greater among those with ASM exposure, our study may have underestimated the association between first-trimester ASM use and miscarriage. Furthermore, among clinically recognisable pregnancies, some early clinical miscarriages may not be reported, which if related to exposure status may have biased our results. Patients with indications of ASMs, or on ASMs, might be more likely to report early pregnancy loss as they have more healthcare use. However, if this were true, we would expect our study to induce a spurious association between first-trimester ASM exposure and miscarriage across all ASM indications, which we did not observe. Also, the mean gestational age among women with and without first-trimester ASM exposure was very similar, suggesting reporting of early pregnancy losses is not greater among ASM users. Furthermore, previous work has demonstrated the CPRD Pregnancy Register has high sensitivity (77%) in identifying hospital-based early pregnancy losses and the overall miscarriage rates in the Pregnancy Register (of 12%–13%) compare favourably with estimates from external sources. Finally, in sensitivity analysis comparing the characteristics of our study cohort to women excluded due to their pregnancy having an unknown outcome, the groups were broadly similar.

Although we identified over 1 million eligible pregnancies, in some analyses the number of ASM exposed pregnancies was small, meaning there may have been insufficient statistical power to detect true associations. For example, the analysis among women with first-trimester ASM exposure included 7807 women, and the comparison of the risks of different ASMs against lamotrigine had wide CIs.

Our exposure groups may be subject to misclassification. First, the CPRD Pregnancy Register estimates pregnancy start from a range of codes (such as estimated date of conception, or LMP) or where codes are unavailable, pregnancy start is singly imputed based on the type of pregnancy outcome. As such, pregnancy start may be misclassified by a matter of weeks or months, leading to misclassification of exposure status. Additionally, the CPRD does not provide information on whether the prescription was dispensed to, or taken by, the patient; as such, some women in our exposed group may be misclassified as exposed. However, when redefining our exposure as two prescriptions in the first trimester and thereby reducing exposure misclassification, the results were similar. As secondary care prescriptions were unavailable, some exposed pregnancies managed in a secondary care setting may have been allocated to the unexposed group. Secondary care ASM prescribing may occur in newly diagnosed patients, where a change in prescriptions is required, or among complex cases not responding to treatment. However, the large majority of ASM prescriptions will occur in primary care with the advice of secondary care specialists. Further, levels of actual ASMs concentration in the body is not recorded in the CPRD, as it would require therapeutic drug monitoring and women experience greater clearance of ASMs during pregnancy, which reduces the ASMs concentration in the blood.^23^ Finally, it is likely women taking ASMs would be advised to taper off their medication rather than abruptly stop; this information is not available in the database, therefore there may be some misclassification of exposure status during the study period.

Finally, there may be residual confounding from variables unavailable in the database. The CPRD does not provide any data on paternal characteristics. Advanced paternal age has been recognised as a risk factor for miscarriage,^24^ which may be associated with maternal ASM exposure status. Parental history of major congenital malformations, another risk factor for miscarriage,^22^ is unlikely to be reliably captured in these data.

This work may help inform women considering taking ASMs during pregnancy or women currently taking ASMs at the point of a confirmed pregnancy. While women taking ASMs had a slightly higher incidence of miscarriage (14.5% vs 12.2% in those without ASM exposure), this work supports existing evidence suggesting ASMs are unlikely to increase the risk of miscarriage. Rather there exists a potential background risk of miscarriage in women who are prescribed these medications due to their underlying condition which may explain previous studies finding an association. While there was an increased risk of miscarriage among women with epilepsy taking high-dose ASMs, in particular for valproate, this association may be driven by confounding by epilepsy severity.

We found no clear evidence to suggest that first-trimester ASM exposure increased the risk of miscarriage. Unmeasured confounding may explain the slight increased risk of miscarriage with first trimester antiseizure drug exposure in women with bipolar or other psychiatric conditions.

## Data Availability

No data are available. This study is based in part on data from the Clinical Practice Research Datalink (CPRD) obtained under licence from the UK Medicines and Healthcare products Regulatory Agency. The terms of our licence to access the data preclude us from sharing individual patient data with third parties. The raw data may be requested directly from CPRD following their usual procedures.

## References

[1] Madley-Dowd P , Rast J , Ahlqvist VH , et al . Trends and patterns of antiseizure medication prescribing during pregnancy between 1995 and 2018 in the United kingdom: a cohort study. BJOG 2024;131:15–25. 10.1111/1471-0528.17573 37340193 PMC10730765

[2] Tomson T , Sha L , Chen L . Management of epilepsy in pregnancy: what we still need to learn. Epilepsy Behav Rep 2023;24:100624. 10.1016/j.ebr.2023.100624 37867487 PMC10585340

[3] Man S-L , Petersen I , Thompson M , et al . Antiepileptic drugs during pregnancy in primary care: a UK population based study. PLoS One 2012;7:e52339. 10.1371/journal.pone.0052339 23272239 PMC3525559

[4] Gedzelman E , Meador KJ . Antiepileptic drugs in women with epilepsy during pregnancy. Ther Adv Drug Saf 2012;3:71–87. 10.1177/2042098611433192 25083227 PMC4110845

[5] Vajda FJE , O’Brien TJ , Graham JE , et al . Antiepileptic drugs, foetal malformations and spontaneous abortions. Acta Neurol Scand 2017;135:360–5. 10.1111/ane.12672 27573510

[6] Viale L , Allotey J , Cheong-See F , et al . Epilepsy in pregnancy and reproductive outcomes: a systematic review and meta-analysis. Lancet 2015;386:1845–52. 10.1016/S0140-6736(15)00045-8 26318519

[7] Margulis AV , Mittleman MA , Glynn RJ , et al . Effects of gestational age at enrollment in pregnancy exposure registries. Pharmacoepidemiol Drug Saf 2015;24:343–52. 10.1002/pds.3731 25702683

[8] Mostacci B , Bisulli F , Poluzzi E , et al . Emilia-Romagna study on pregnancy and exposure to antiepileptic drugs (ESPEA): a population-based study on prescription patterns, pregnancy outcomes and fetal health. J Neurol Neurosurg Psychiatry 2018;89:983–8. 10.1136/jnnp-2017-317833 29549194 PMC6109238

[9] Richards N , Reith D , Stitely M , et al . Antiepileptic drug exposure in pregnancy and pregnancy outcome from national drug usage data. BMC Pregnancy Childbirth 2018;18:84. 10.1186/s12884-018-1728-y 29625554 PMC5889580

[10] Hernández-Díaz S , Smith CR , Shen A , et al . Comparative safety of antiepileptic drugs during pregnancy. Neurology 2012;78:1692–9. 10.1212/WNL.0b013e3182574f39 22551726

[11] Trivedi M , Jose M , Philip RM , et al . Spontaneous fetal loss in women with epilepsy: prospective data from pregnancy registry in India. Epilepsy Res 2018;146:50–3. 10.1016/j.eplepsyres.2018.07.016 30077056

[12] Vajda FJE , O’Brien TJ , Graham J , et al . Anti-epileptic drug exposure and risk of foetal death in utero. Acta Neurol Scand 2018;137:20–3. 10.1111/ane.12816 28857118

[13] Herrett E , Gallagher AM , Bhaskaran K , et al . Data resource profile: clinical practice research datalink (CPRD). Int J Epidemiol 2015;44:827–36. 10.1093/ije/dyv098 26050254 PMC4521131

[14] Chisholm J . The read clinical classification. BMJ 1990;300:1092. 10.1136/bmj.300.6732.1092 2344534 PMC1662793

[15] Minassian C , Williams R , Meeraus WH , et al . Methods to generate and validate a pregnancy register in the UK clinical practice research datalink primary care database. Pharmacoepidemiol Drug Saf 2019;28:923–33. 10.1002/pds.4811 31197928 PMC6618019

[16] Campbell J , Bhaskaran K , Thomas S , et al . Investigating the optimal handling of uncertain pregnancy episodes in the CPRD GOLD pregnancy register: a methodological study using UK primary care data. BMJ Open 2022;12:e055773. 10.1136/bmjopen-2021-055773 PMC8867343 35193920

[17] Petersen AH , Lange T . What is the causal interpretation of sibling comparison designs? Epidemiology 2020;31:75–81. 10.1097/EDE.0000000000001108 31651661

[18] Sjölander A , Frisell T , Kuja-Halkola R , et al . Carryover effects in sibling comparison designs. Epidemiology 2016;27:852–8. 10.1097/EDE.0000000000000541 27488059

[19] Bech BH , Kjaersgaard MIS , Pedersen HS , et al . Use of antiepileptic drugs during pregnancy and risk of spontaneous abortion and stillbirth: population based cohort study. BMJ 2014;349:g5159. 10.1136/bmj.g5159 25150301 PMC4141333

[20] Veroniki AA , Cogo E , Rios P , et al . Comparative safety of anti-epileptic drugs during pregnancy: a systematic review and network meta-analysis of congenital malformations and prenatal outcomes. BMC Med 2017;15:95. 10.1186/s12916-017-0845-1 28472982 PMC5418725

[21] Mostacci B , Poluzzi E , D’Alessandro R , et al . Adverse pregnancy outcomes in women exposed to gabapentin and pregabalin: data from a population-based study. J Neurol Neurosurg Psychiatry 2018;89:223–4. 10.1136/jnnp-2017-316143 28716783

[22] Tomson T , Battino D , Bonizzoni E , et al . Antiepileptic drugs and Intrauterine death: a prospective observational study from EURAP. Neurology 2015;85:580–8. 10.1212/WNL.0000000000001840 26187231

[23] Pennell PB , Karanam A , Meador KJ , et al . Antiseizure medication concentrations during pregnancy: results from the maternal outcomes and neurodevelopmental effects of antiepileptic drugs (MONEAD) study. JAMA Neurol 2022;79:370–9. 10.1001/jamaneurol.2021.5487 35157004 PMC8845026

[24] du Fossé NA , van der Hoorn M-LP , van Lith JMM , et al . Advanced paternal age is associated with an increased risk of spontaneous miscarriage: a systematic review and meta-analysis. Hum Reprod Update 2020;26:650–69. 10.1093/humupd/dmaa010 32358607 PMC7456349

