# Supplementary material for: First-trimester use of antiseizure medications and the risk of miscarriage: a population-based cohort study
Source: J Neurol Neurosurg Psychiatry. 2024 May 22;95(8):693–703. doi: 10.1136/jnnp-2023-333149 (PMC11287565; doi:10.1136/jnnp-2023-333149)
Supplement: Supplementary data [file jnnp-2023-333149supp001.pdf]

Supplementary material

Supplementary methods:

Methods S1: Uncertain pregnancies in the CPRD Pregnancy Register

A substantial proportion of pregnancies in the CPRD GOLD Pregnancy Register are uncertain, either having no identified outcome, or they overlap ("conflict") with other pregnancies. Ignoring these records potentially excludes periods when women were pregnant. Work by the CPRD Pregnancy Register developers[16] has investigated the frequency of various scenarios explaining unknown/conflicting pregnancies, and in line with their advice for drug safety studies we performed the following actions:

1. Utilise linked data to obtain additional outcomes

The CPRD Pregnancy Register only utilises data from primary care record. We used HES data (HES APC, HES Maternity, HES Outpatients and HES Procedures) to identify outcomes (delivery, miscarriage, or termination of pregnancy). For deliveries, records within 266 days of pregnancy start plus 4 weeks (38 weeks) were retained, and for early pregnancy losses, records within 140 days from first antenatal record (20 weeks) were retained. HES records were only available for those with linked data (N=596,218 (48.5%)). We used the approach outlined in the CPRD Pregnancy Register validation study.[25]

2. Merging conflicting pregnancies episodes

We merged conflicting episodes which are consistent with the pregnancy being real, but split into separate episodes by the rules of the Pregnancy Register algorithm.

3. Excluding episodes which are likely to be derived from historical data

There is evidence to suggest that historical outcomes being recorded by the GP during an ongoing pregnancy may explain a sizeable proportion of the uncertain episodes generated by the algorithm. This can lead to true pregnancies being split by the algorithm and depending on the timing this will either generate an additional episode with outcome missing or two separate episodes with outcomes.

Methods S2: ASM prescriptions: further detail on data cleaning procedures

Cleaning procedure applied to the ASM prescriptions

Implausible values for number of tablets taken per day and total quantity of tablets prescribed were changed to missing, and a hot-decking approach was used to singly impute missing values for quantity and number of tablets taken per day, using similar prescriptions first within the individual and then similar individuals.[26] Prescription length was calculated by dividing the quantity of tablets by the number taken per day.

We used an imputation approach called "Hot Decking" to address missing total quantity of tablets prescribed and number of tablets taken per day (referred to as DD). Hot Decking involves replacing missing data with observed data from a similar unit, or strata, for example patients of the same age and gender. The modal numeric daily dose of the observed data, within the chosen strata, was used to replace missing numeric daily dose within that same strata. An algorithm was developed which reviewed each therapy record and imputed missing values, first where the strata were based on data within- persons, then where the strata were based on data from groups of patients.

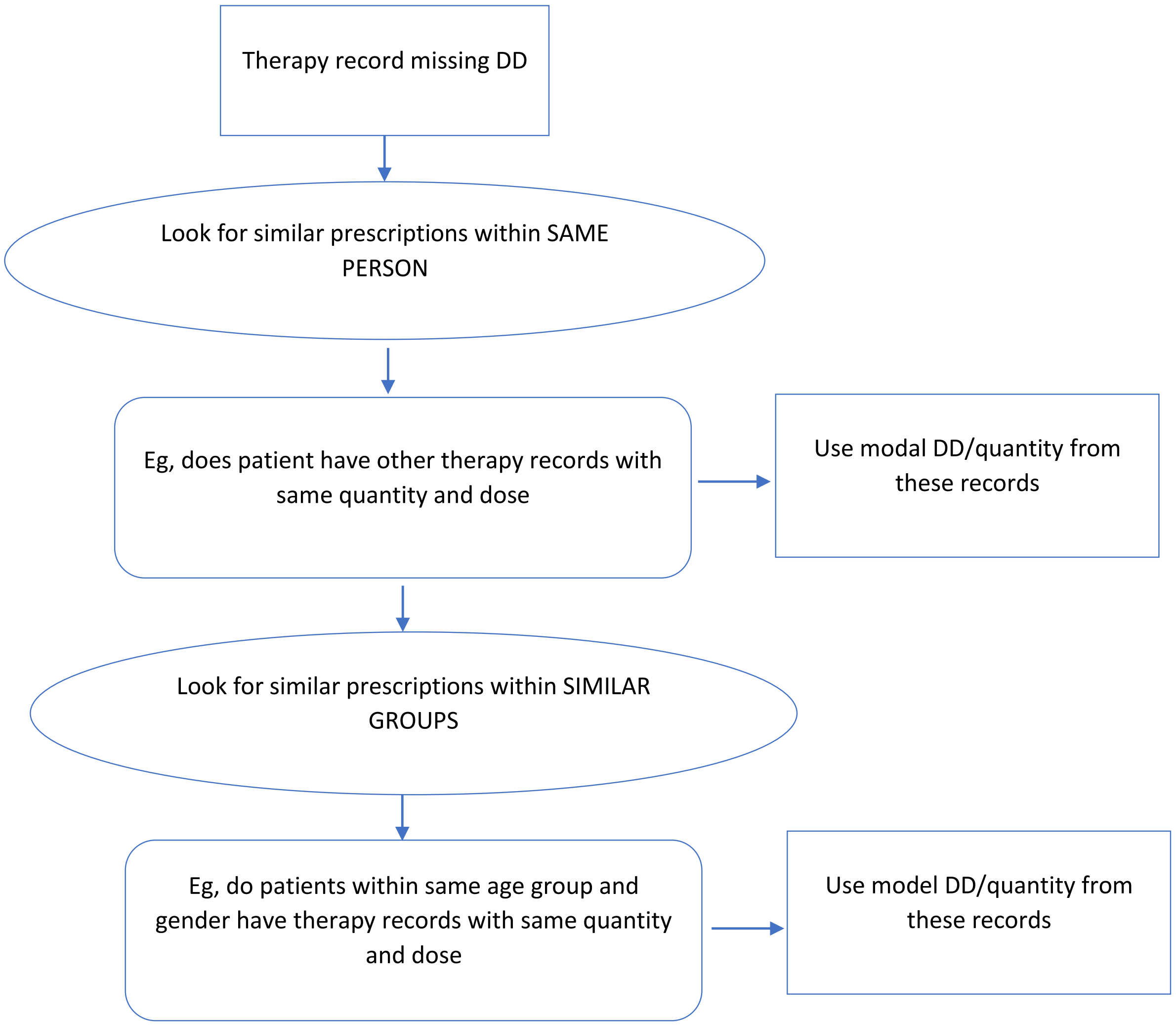

We considered women exposed to a drug in any period if the length of a prescription for the drug overlapped with that time period.

Dose Distribution Within Each Dose Range Category for all Antiseizure prescriptions

We classified each prescription as low, medium and high dose. The cut-offs for these dose categories were developed from all pre-pregnancy prescriptions; we calculated quartiles of distributions of daily doses in milligrams, separately for each individual drug, and then combined this information in a single dose level variable (1st quartile defined as low doses, 2nd and third quartiles as moderate doses, 4th quartile as high doses). See the table below for cut-offs for each ASM drug type based on this approach. Daily dose for the first trimester was then identified, using these cut-offs. In case of pregnancies where prescriptions had been issued at different dose levels during the same trimester, we used the highest daily dose prescribed.

Polytherapy

Polytherapy was defined as having prescriptions of two or more ASMs during the first trimester. Where women have prescriptions for more than one type of drug on different days, we classified them according to the drug class prescribed first.

ASM type

In the “other” ASM type category, the ASMs were: brivaracetam, eslicarbazepine, ethosuximide, felbamate, lacosamide, oxcarbazepine, perampanel, phenobarbital, primidone, retigabine, rufinamide, stiripentol, sulthiame, tiagabine, vigabatrin, zonisamide, beclamide, mesuximide, phenacemide, ethotoin, pheneturide, carisbamate, cenobamate, barbexaclone, ethadione, progabide, clobazam.

BMJ Publishing Group Limited (BMJ) disclaims all liability and responsibility arising from any reliance placed on this supplemental material which has been supplied by the author(s)

Drug	Total Rx	Level	Value (mg/day)	N	Minimum	25% QI	50% Median	75% Q3	Maximium
carbamazepine	18534	Low	<=300	724	50	200	200	200	300
		Medium	>300 to <=800	1658	400	400	600	800	800
		High	>800	268	900	1200	1200	1600	480000
lamotrigine	27370	Low	<=100	970	10	50	100	100	100
		Medium	>100 to <=200	1111	125	200	200	200	200
		High	>200	610	225	400	400	400	80000
phenobarbital	595	Low	<=60	29	15	30	60	60	60
		Medium	>60 to <=180	19	90	90	120	120	180
		High	>180	15	210	300	500	500	1500
valproate	15104	Low	<=600	772	100	400	500	600	600
		Medium	>600 to <=1200	956	700	800	1000	1000	1200
		High	>1200	421	1250	1500	2000	2000	500000
brivaracetam	17	Low	<=200	1	200	200	200	200	200
		Medium	>200 to <=200	0	700	800	1000	1000	1200
		High	>200	0	1250	1500	2000	2000	500000
eslicarbazepine	88	Low	<=800	4	800	800	800	800	800
		Medium	>800 to <=1200	4	1200	1200	1200	1200	1200
		High	>1200	2	1600	1600	1600	1600	1600
ethosuximide	305	Low	<=750	12	250	500	500	750	750
		Medium	>750 to <=1500	22	1000	1000	1000	1250	1500
		High	>1500	8	2000	2000	3500	5000	5000
gabapentin	13643	Low	<=300	819	100	300	300	300	300
		Medium	>300 to <=1200	1564	400	900	900	900	1200
		High	>1200	742	1350	1800	1800	2400	540000
lacosamide	276	Low	<=200	12	100	150	200	200	200
		Medium	>200 to <=400	15	300	300	300	400	400
		High	>400	1	600	600	600	600	600
levetiracetam	8150	Low	<=1000	368	250	750	1000	1000	1000
		Medium	>1000 to <=2000	373	1250	1500	2000	2000	2000
		High	>2000	114	2250	3000	3000	4000	500000
oxcarbazepine	205	Low	<=600	12	300	600	600	600	600
		Medium	>600 to <=1200	10	750	1200	1200	1200	1200
		High	>1200	3	1800	1800	1800	1800	1800
perampanel	47	Low	<=6	2	2	2	4	6	6

		Medium	>6 to <=8	2	8	8	8	8	8
		High	>8	1	12	12	12	12	12
phenytoin	2295	Low	<=200	92	25	100	200	200	200
		Medium	>200 to <=300	116	300	300	300	300	300
		High	>300	46	400	400	400	400	90000
pregabalin	12634	Low	<=150	1039	21.42857	75	150	150	150
		Medium	>150 to <=300	484	200	225	300	300	300
		High	>300	411	375	450	600	600	270000
primidone	59	Low	<=500	7	500	500	500	500	500
		Medium	>500 to <=500	0	200	225	300	300	300
		High	>500	1	1500	1500	1500	1500	1500
retigabine	6	Low	<=600	1	600	600	600	600	600
		Medium	>600 to <=600	0	200	225	300	300	300
		High	>600	0	1500	1500	1500	1500	1500
rufinamide	0								
stiripentol	42	Low	<=500	1	500	500	500	500	500
		Medium	>500 to <=500	0	200	225	300	300	300
		High	>500	0	1500	1500	1500	1500	1500
tiagabine	31	Low	<=15	1	10	10	10	10	10
		Medium	>15 to <=32.5	2	20	20	20	20	20
		High	>32.5	1	45	45	45	45	45
topiramate	5087	Low	<=50	397	15	30	50	50	50
		Medium	>50 to <=100	309	60	100	100	100	100
		High	>100	186	125	200	200	400	1875
vigabatrin	188	Low	<=1250	6	1000	1000	1000	1000	1250
		Medium	>1250 to <=4000	16	1500	2000	3000	4000	4000
		High	>4000	0	125	200	200	400	1875
zonisamide	470	Low	<=100	21	25	50	100	100	100
		Medium	>100 to <=200	22	150	200	200	200	200
		High	>200	13	300	300	400	400	500
clonazepam	2427	Low	<=.5	100	0.25	0.5	0.5	0.5	0.5
		Medium	>.5 to <=2	209	0.75	1	1	2	2
		High	>2	45	2.5	3	4	6	16

Methods S3: Detailed definitions of indications: women could have more than one indication

**Indication**	**Derivation**
**Epilepsy**	One of the following:<br>• Diagnosis of epilepsy according to the pre-specified algorithm (see Figure S1), OR;<br>• Epilepsy-specific ASMSs: Epilim, Brivaracetam, Brivaracetam, Eslicarbazepine, Ethosuximide, Felbamate, Fenfluramine, Lacosamide, Levetiracetam, Mesuximide, Oxcarbazepine, Perampanel, Phenobarbital, Phenytoin, Retigabine, Rufinamide, Stiripentol, Sulthiame, Tiagabine, Vigabatrin, Zonisamide, OR;<br>• Epilepsy-specific co-prescribing on the same day: 1) Clobazam and an ASMS or 2) rectal administration of diazepam and an ASMS or 3) intranasal administration of Midazolamand ASMS
**Bipolar disorder**	One of the following:<br>• Read code in CPRD or ICD-10 code in HES (any diagnostic field) for bipolar, anytime prior to pregnancy start date OR;<br>• Mood-disorder specific co-prescribing (1-Quetiapine and [valproate or lamotrigine or carbamazepine] or 2-lithium and [valproate or lamotrigine or carbamazepine]) OR;<br>• The mood disorder-specific ASMS Depakote.
**Other somatic conditions**	
Neuropathic pain (including diabetic neuropathy) and fibromyalgia	A READ code in CPRD or ICD-10 code for a neuropathic pain disorder, anytime prior to pregnancy start.
Migraine prophylaxis	AREAD code in CPRD or ICD-10 code for migraines, anytime prior to pregnancy start.
Restless legs syndrome	a READ code for restleg leg syndrome in CPRD , anytime prior to pregnancy start.
Essential tremors	h A READ code for essential tremors in CPRD , anytime prior to pregnancy start.
**Other psychiatric conditions**	
Generalised anxiety disorder	A Read code in CPRD on the same day, anytime prior to pregnancy start date.
Depression and other affective disorders	A READ code in CPRD for depression , anytime prior to pregnancy start.
schizophrenia/ /psychosis	A READ code in CPRD for schizophrenia, anytime prior to pregnancy start.
Other off-label psychiatric use	Where none of the above indications were identified, yet there was prescription of antipsychotics, lithium, or antidepressants

Methods S4: Description of sensitivity analyses conducted

Possible bias	Analysis	Description
**Confounding by history of pregnancy loss**	*Restriction to first pregnancies*	To account for potential residual confounding by history of pregnancy loss, we repeated the main analyses, restricted the first pregnancy for each woman in the CPRD Pregnancy Register.
**Exposure misclassification**	*Redefine primary exposure as two ASM prescriptions in first trimester*	To be confident a patient is using medication and has not discontinued medication prior to pregnancy, we required the exposed group to have two ASM prescriptions of the same type in the first trimester. The majority of ASM prescriptions in the UK are 28 days, meaning women using ASMs consistently should require at least three prescriptions during their first trimester.
	Splitting exposed group into new and prevalent users	To explore whether the risk of first trimester exposure differed according to whether the woman was a new or prevalent user, we split the main exposure group into new and prevalent users, where "new users" were those women without a prescription in the previous 12 months and "prevalent users" were those with use in the previous 12 months.
**Outcome misclassification**	*Restricting to women with linked HES data*	To explore whether there was under-ascertainment of miscarriage, we restricted to patients with linked HES data, where we were able to supplement the CPRD pregnancy register with hospital records of miscarriage.
	Comparison of included pregnancies with those excluded due to missing outcome	In line with recommendations from the CPRD Pregnancy Register developers, we did not carry out a multiple imputation analysis on pregnancies with unknown outcome, as there are not sufficient predictors of the missing pregnancy outcome in the dataset.[16] As such, we compared the characteristics of women include in the cohort, and those women excluded due to their pregnancy outcome being unknown.

Figure S1: Flow diagrams to identify patients with epilepsy in CPRD and their linked datasets

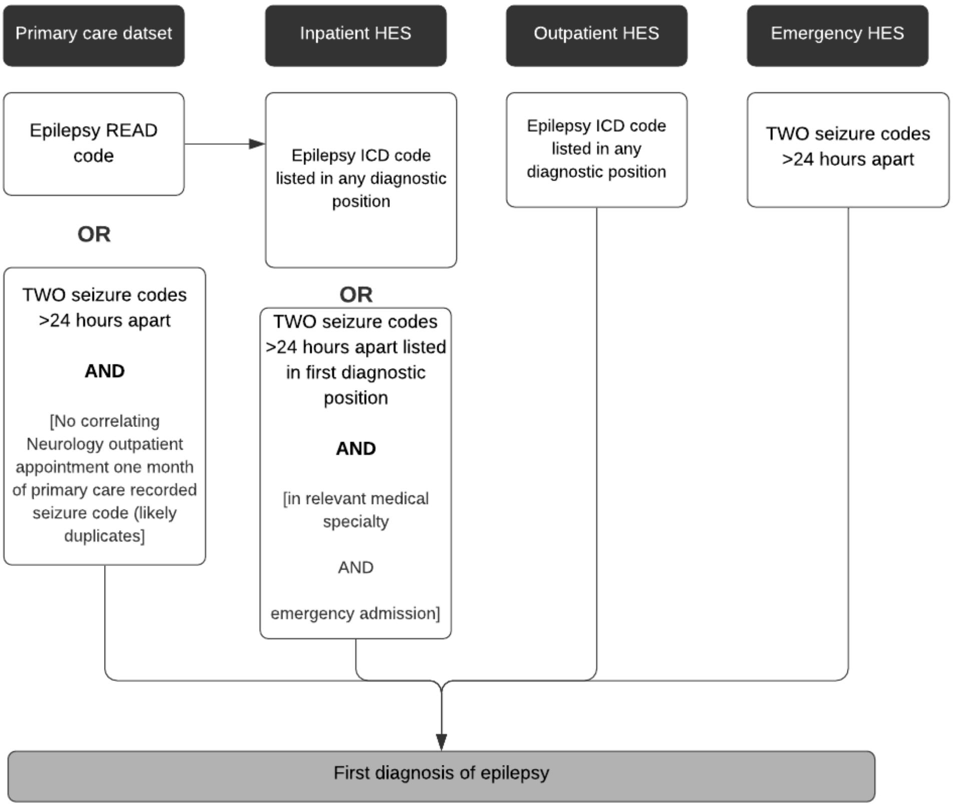

Table S1: Number of miscarriages, total pregnancies and proportion with miscarriages, in exposed and unexposed and adjusted HRs of miscarriage associated with antiseizure medications treatment in first trimester of pregnancy, overall and stratified by ASM indication.

**Indication**	**Comparison group**	**N miscarriages**	**Total person years**	**Rate per 10,000 person years**	**Total pregnancies**	**Percent**	**Unadjusted HR (95% CI)**	**Fully adjusted* HR (95% CI)**	**Fully adjusted* + ethnicity HR (95% CI)**
**All women**	Unexposed	124285	388989.1	31.95	1016535	12.23	1.00 (ref)	1.00 (ref)	1.00 (ref)
	First trimester exposed	1139	2932.9	38.84	7832	14.54	1.20 (1.13-1.27)	1.06 (1.00-1.13)	1.05 (0.97-1.13)
**Epilepsy**	Unexposed	1133	3219.97	35.19	8593	13.19	1.00 (ref)	1.00 (ref)	1.00 (ref)
	First trimester exposed	622	1804.01	34.48	4725	13.16	0.99 (0.89-1.09)	0.98 (0.89-1.08)	0.97 (0.86-1.08)
**Bipolar and other psychiatric**	Unexposed	49222	137596.7	35.77	365484	13.47	1.00 (ref)	1.00 (ref)	1.00 (ref)
	First trimester exposed	787	1845.69	42.64	5002	15.73	1.18 (1.10-1.26)	1.08 (1.00-1.16)	1.07 (0.98-1.17)
**Other somatic**	Unexposed	18163	50446.56	36	132354	13.72	1.00 (ref)	1.00 (ref)	1.00 (ref)
	First trimester exposed	360	862.9	41.72	2313	15.56	1.14 (1.03-1.26)	1.04 (0.93-1.16)	1.05 (0.92-1.19)

*Adjusted for: maternal age, year of pregnancy start, IMD, history of pregnancy loss, epilepsy, bipolar, other psychiatric conditions, other somatic conditions.

Table S2: Post-hoc analysis to investigate exposure discordant pregnancies

Indication		Main analysis: women with at least two pregnancies where at least two were exposure discordant			Restricted dataset of 1st two pregnancies in cohort (Restricted cohort)			Discordant sample restricted to women who took ASMs in the second pregnancy (Restricted cohort 2).			Discordant sample restricted to women who took ASMs in the first pregnancy (Restricted cohort 3)		
		Total N	Number (%) miscarriages	HR (95% CI)	Total N	Number (%) miscarriages	HR (95% CI)	Total N	Number (%) miscarriages	HR (95% CI)	Total N	Number (%) miscarriages	HR (95% CI)
**All women**	Unexposed	3216	450 (14.0)	1.00 (ref)	2320	335 (14.4)	1.00 (ref)	1993	289(68.5)	1.00 (ref)	327	46 (33.8)	1.00 (ref)
	Exposed	2039	381 (18.7)	1.28 (1.10-1.49)	1124	223 (19.8)	1.19 (0.94-1.52)	713	133 (31.5)	0.71 (0.48-1.05)	411	90 (66.2)	2.33 (1.38-3.95)
**Women with epilepsy**	Unexposed	523	63 (12.1)	1.00 (ref)	318	37 (11.6)	1.00 (ref)	168	24 (38.1)	1.00 (ref)	150	13 (22.0)	1.00 (ref)
	Exposed	712	132 (18.5)	1.47 (1.03-2.09)	429	85 (19.8)	1.93 (1.10-3.37)	221	39 (61.9)	0.52 (0.13-2.08)	208	46 (78.0)	3.85 (1.44-10.30)
**Women with bipolar or other psychiatric disorders**	Unexposed	2253	319 (14.2)	1.00 (ref)	1521	230 (15.1)	1.00 (ref)	1268	190 (64.2)	1.00 (ref)	253	40 (41.2)	1.00 (ref)
	Exposed	1628	303 (18.6)	1.23 (1.04-1.46)	851	163 (19.2)	1.09 (0.82-1.46)	589	106 (35.8)	0.62 (0.38-1.02)	262	57 (58.8)	1.91 (1.04-3.50)
**Women with other somatic conditions**	Unexposed	931	141 (15.2)	1.00 (ref)	568	94 (16.6)	1.00 (ref)	455	76 (58.5)	1.00 (ref)	113	18 (40.9)	1.00 (ref)
	Exposed	792	140 (17.7)	1.22 (0.94-1.58)	418	80 (19.1)	0.83 (0.52-1.31)	284	54 (41.5)	0.35 (0.15-0.82)	134	26 (59.1)	2.58 (1.16-5.75)

*****Adjusted for: age, IMD, epilepsy, bipolar, other psychiatric conditions, other somatic conditions.

To test whether the results from the discordant exposure analyses were susceptible to the influence of carry-over effects,[18] in this case where the outcome of the first pregnancy influences exposure in the second pregnancy, we performed a post-hoc sensitivity analysis. First, we restricted the sample to include only first two pregnancies for each woman. Analyses were run on this dataset as a whole (restricted cohort) to confirm comparability with our main analyses when using only the first two children in a family. We then repeated the analysis, restricting the exposure discordant group to those in which the woman discontinued ASMs in the first pregnancy and took ASMs in the second pregnancy (restricted cohort 2) and then repeated with further restriction of the exposure discordant group to only those in which the woman took ASMs in the first pregnancy and discontinued ASM use in the second pregnancy (restricted cohort 3). In the absence of 'asymmetrical' carry-over effects[18] we would expect similar HR estimates with value greater than 1 in both restricted cohort 2 and 3. Instead we see elevated HRs when mothers are exposed in the first pregnancy (restricted cohort 3) but not when mothers are exposed in the second pregnancy (restricted cohort 2). We interpret this to mean that the increased hazard of miscarriage following exposure to ASMs in exposure discordant pregnancy analyses could be explained by the ordering of which pregnancy was exposed and not necessarily the result of the ASM itself.

Table S3: Number of events, crude event rates (per 1000), crude IRRs and adjusted HRs for miscarriage to the dose during pregnancy**, among those with any antiseizure medications treatment in first trimester.**

Indication	ASM type	Dose	N events	%	Unadjusted HR	Fully adjusted HR* (95% CI)
**All**	Lamotrigine (N=1916)	Low	84	13.31	1.00 (ref)	1.00 (ref)
		Medium	109	13.26	1.01 (0.76-1.33)	1.00 (0.76-1.31)
		High	60	12.96	0.97 (0.69-1.38)	0.99 (0.71-1.39)
	Carbamazepine (N=1523)	Low	43	14.88	1.00 (ref)	1.00 (ref)
		Medium	155	14.72	1.01 (0.74-1.39)	1.16 (0.84-1.59)
		High	29	16.02	1.07 (0.70-1.63)	1.31 (0.86-2.00)
	Gabapentin (N=1224)	Low	41	15.53	1.00 (ref)	1.00 (ref)
		Medium	98	16.42	1.03 (0.72-1.47)	1.01 (0.70-1.46)
		High	62	17.08	1.06 (0.72-1.54)	1.07 (0.73-1.57)
	Valproate (N=1109)	Low	42	10.77	1.00 (ref)	1.00 (ref)
		Medium	65	13	1.23 (0.82-1.84)	1.19 (0.81-1.77)
		High	34	15.53	1.49 (0.94-2.37)	1.49 (0.94-2.36)
**Epilepsy**	Lamotrigine (N=1656)	Low	59	11.92	1.00 (ref)	1.00 (ref)
		Medium	92	12.81	1.10 (0.79-1.52)	1.07 (0.78-1.46)
		High	57	12.87	1.09 (0.74-1.61)	1.05 (0.73-1.51)
	Carbamazepine (N=1193)	Low	16	11.35	1.00 (ref)	1.00 (ref)
		Medium	125	14.16	1.28 (0.79-2.07)	1.26 (0.81-1.95)
		High	26	15.38	1.36 (0.78-2.40)	1.42 (0.83-2.42)
	Gabapentin (N=75)	Low	1	16.67	1.00 (ref)	1.00 (ref)
		Medium	4	11.76	0.85 (0.15-4.87)	0.59 (0.03-12.57)
		High	6	17.14	1.06 (0.21-5.42)	0.93 (0.05-19.11)
	Valproate (N=930)	Low	27	8.88	1.00 (ref)	1.00 (ref)
		Medium	56	13.15	1.53 (0.92-2.53)	1.39 (0.84-2.29)
		High	32	16	1.89 (1.09-3.28)	1.72 (1.00-2.96)
**Bipolar and other psychiatric**	Lamotrigine (N=983)	Low	50	14.62	1.00 (ref)	1.00 (ref)
		Medium	56	14.21	0.98 (0.67-1.41)	0.99 (0.69-1.41)
		High	36	14.57	0.97 (0.63-1.50)	0.98 (0.64-1.50)
	Carbamazepine (N=764)	Low	28	16.37	1.00 (ref)	1.00 (ref)
		Medium	89	17.91	1.11 (0.74-1.65)	1.30 (0.87-1.96)
		High	19	19.79	1.21 (0.72-2.02)	1.65 (0.97-2.82)
	Gabapentin (N=1105)	Low	39	17.26	1.00 (ref)	1.00 (ref)
		Medium	85	15.86	0.89 (0.62-1.28)	0.86 (0.59-1.24)
		High	59	17.2	0.95 (0.65-1.40)	0.95 (0.65-1.39)
	Valproate (N=589)	Low	30	14.63	1.00 (ref)	1.00 (ref)
		Medium	37	13.96	0.96 (0.59-1.56)	0.92 (0.57-1.48)
		High	18	15.13	1.11 (0.62-1.96)	1.04 (0.59-1.85)
**Other somatic**	Lamotrigine (N=371)	Low	19	16.67	1.00 (ref)	1.00 (ref)
		Medium	19	13.29	0.87 (0.47-1.60)	0.90 (0.47-1.71)
		High	18	15.79	0.99 (0.51-1.91)	1.12 (0.56-2.26)
	Carbamazepine (N=325)	Low	14	14.89	1.00 (ref)	1.00 (ref)
		Medium	30	15.31	1.01 (0.55-1.86)	1.65 (0.93-2.91)
		High	6	17.14	1.06 (0.43-2.62)	1.99 (0.81-4.87)
	Gabapentin (N=565)	Low	21	17.95	1.00 (ref)	1.00 (ref)
		Medium	41	14.39	0.80 (0.49-1.31)	0.79 (0.48-1.31)
		High	29	17.79	0.96 (0.57-1.62)	0.98 (0.57-1.68)
	Valproate (N=216)	Low	11	12.09	1.00 (ref)	1.00 (ref)
		Medium	8	9.2	0.80 (0.29-2.24)	0.84 (0.33-2.17)
		High	3	7.89	0.66 (0.17-2.66)	0.76 (0.18-3.18)

Table S4: Results from sensitivity analysis

Sensitivity Analysis	Indication	Exposure	N miscarriages	N total pregnancies	Percent	Unadjusted HR (95% CI)	Fully adjusted* HR (95% CI)
**Restricted to linked data**	All women	Unexposed	63076	524954	12.01553	1.00 (ref)	1.00 (ref)
		Exposed	497	3391	14.65644	1.20 (1.10-	1.10 (1.00-1.22)
	Women with epilepsy	Unexposed	585	4609	12.69256	1.00 (ref)	1.00 (ref)
		Exposed	309	2255	13.70288	1.05 (0.91-	1.07 (0.93-1.23)
	Women with bipolar or other psychiatric disorders	Unexposed	24418	183406	13.31363	1.00 (ref)	1.00 (ref)
		Exposed	330	2021	16.32855	1.19 (1.07-	1.16 (1.03-1.30)
	Women with other somatic conditions	Unexposed	9632	71084	13.55017	1.00 (ref)	1.00 (ref)
		Exposed	176	1018	17.2888	1.26 (1.09-	1.19 (1.02-1.39)
**Restricted to first pregnancies**	All women	Unexposed	34989	322566	10.84708	1.00 (ref)	1.00 (ref)
		Exposed	288	2303	12.50543	1.10 (0.98-	1.01 (0.89-1.15)
	Women with epilepsy	Unexposed	283	2437	11.61264	1.00 (ref)	1.00 (ref)
		Exposed	162	1513	10.7072	0.87 (0.72-	0.88 (0.73-1.06)
	Women with bipolar or other psychiatric disorders	Unexposed	10428	84063	12.40498	1.00 (ref)	1.00 (ref)
		Exposed	170	1195	14.22594	1.08 (0.93-	1.04 (0.88-1.22)
	Women with other somatic conditions	Unexposed	4109	33424	12.29356	1.00 (ref)	1.00 (ref)
		Exposed	81	571	14.18564	1.10 (0.89-	1.07 (0.85-1.35)
**Require two prescriptions to be classified as exposed**	All women	Unexposed	124285	1016144	12.23104	1.00 (ref)	1.00 (ref)
		Exposed	850	5978	14.2188	1.10 (1.03-	1.02 (0.95-1.10)
	Women with epilepsy	Unexposed	1133	8543	13.26232	1.00 (ref)	1.00 (ref)
		Exposed	525	4022	13.05321	0.94 (0.85-	0.95 (0.86-1.05)
	Women with bipolar or other psychiatric disorders	Unexposed	49222	365183	13.47872	1.00 (ref)	1.00 (ref)
		Exposed	566	3658	15.47294	1.07 (0.99-	1.04 (0.95-1.13)
	Women with other somatic conditions	Unexposed	18163	132213	13.73768	1.00 (ref)	1.00 (ref)
		Exposed	252	1637	15.39401	1.07 (0.95-	1.01 (0.89-1.15)
**Split exposed into new and prevalent users**	All women	Unexposed	124290	1016560	12.22653	1.00 (ref)	1.00 (ref)
		Exposed - prevalent	1038	7227	14.36281	1.18 (1.11-	1.04 (0.97-1.11)
		Exposed - new user	96	580	16.55172	1.41 (1.17-	1.25 (1.04-1.51)
	Women with epilepsy	Unexposed	1136	8610	13.19396	1.00 (ref)	1.00 (ref)
		Exposed - prevalent	611	4624	13.21367	0.98 (0.89-	0.98 (0.89-1.08)
		Exposed - new user	8	84	9.523809	0.78 (0.40-	0.84 (0.43-1.63)
	Women with bipolar or other	Unexposed	49225	365499	13.46789	1.00 (ref)	1.00 (ref)

	psychiatric disorders	Exposed - prevalent	705	4544	15.51497	1.15 (1.07-	1.05 (0.97-1.14)
		Exposed - new user	79	443	17.83296	1.37 (1.12-	1.29 (1.05-1.58)
	Women with other somatic conditions	Unexposed	18164	132362	13.72297	1.00 (ref)	1.00 (ref)
		Exposed - prevalent				. (.-.)	. (.-.)
		Exposed - new user					

*Adjusted for: age, IMD, year of pregnancy, history of pregnancy loss, epilepsy, bipolar, other psychiatric conditions, other somatic conditions.

Table S5: Maternal characteristics at start of pregnancy: comparison of those included in the cohort with those excluded due to unknown outcome.

	**Pregnancies included, N(%)**	**Pregnancies excluded due to outcome unknown, N(%)**
**Total**	1023787 (100.0)	159765 (100.0)
**Exposed first trimester**	7832 (0.8)	1490 (0.9)
**Age in years**		
<18	38216 (3.7)	6690 (4.2)
18-24	231835 (22.6)	39188 (24.5)
25-29	265563 (25.9)	39395 (24.7)
30-34	285357 (27.9)	38694 (24.2)
>=35	202816 (19.8)	35798 (22.4)
**IMD quintile**		
1 (least deprived)	194057 (19.0)	26804 (16.8)
2	178762 (17.5)	25001 (15.6)
3	196900 (19.2)	33026 (20.7)
4	208567 (20.4)	34730 (21.7)
5 (most deprived)	245501 (24.0)	40204 (25.2)
**Ethnicity**		
White	645744 (63.1)	79191 (49.6)
South Asian	32858 (3.2)	4997 (3.1)
Black	17869 (1.7)	3650 (2.3)
Other	11580 (1.1)	1941 (1.2)
Mixed	6950 (0.7)	1164 (0.7)
Not Stated	308786 (30.2)	68822 (43.1)
**Smoking status**		
Non-smoker	415837 (40.6)	65191 (40.8)
Current smoker	307607 (30.0)	50499 (31.6)
Ex-smoker	245838 (24.0)	33144 (20.7)
Not stated	54505 (5.3)	10931 (6.8)
**BMI**		
Underweight	32972 (3.2)	5440 (3.4)
Normal weight	463975 (45.3)	69908 (43.8)
Overweight	239081 (23.4)	35119 (22.0)
Obese	182915 (17.9)	27340 (17.1)
Not stated	104844 (10.2)	21958 (13.7)
**Year of pregnancy start**		
1995-2000	136079 (13.3)	21080 (13.2)
2001-2005	245206 (24.0)	32626 (20.4)
2006-2010	305563 (29.8)	44571 (27.9)
2011-2015	249123 (24.3)	43245 (27.1)
2016-2018	87816 (8.6)	18243 (11.4)

**Problem drinking**	10176 (1.0)	1740 (1.1)
**Illicit drug use**	2294 (0.2)	253 (0.2)
**Consultations ***		
0	83181 (8.1)	15702 (9.8)
1-3	271949 (26.6)	39535 (24.7)
4-10	439136 (42.9)	64566 (40.4)
>10	229521 (22.4)	39962 (25.0)
**Other ASM indications**		
Epilepsy	13234 (1.3)	2250 (1.4)
Bipolar or other psychiatric	370043 (36.1)	58558 (36.7)
Other somatic	134475 (13.1)	19759 (12.4)
**Other prescriptions***		
Antipsychotics	808 (0.1)	163 (0.1)
Antidepressants	114288 (11.2)	20984 (13.1)
Multivitamins	603 (0.1)	63 (0.0)
Folic acid	268760 (26.3)	30513 (19.1)
**Comorbidities**		
Asthma	171174 (16.7)	25977 (16.3)
CKD	3829 (0.4)	655 (0.4)
Diabetes	7343 (0.7)	1530 (1.0)
**Pregnancy history**		
**Gravidity**		
0	0 (0.0)	0 (0.0)
1	547263 (53.5)	0 (0.0)
>=2	160526 (15.7)	0 (0.0)
**Previous miscarriage**	100302 (9.8)	8773 (5.5)
